# Feasibility of an Online Delivered, Home-Based Resistance Training Program for Older Adults – A Mixed Methods Approach

**DOI:** 10.3389/fpsyg.2022.869573

**Published:** 2022-06-03

**Authors:** Sanna Vikberg, Sabine Björk, Anna Nordström, Peter Nordström, Andreas Hult

**Affiliations:** ^1^Department of Health Sciences, Swedish Winter Sports Research Centre, Mid Sweden University, Östersund, Sweden; ^2^Department of Public Health and Clinical Medicine, Section of Sustainable Health, Umeå University, Umeå, Sweden; ^3^Department of Nursing, Umeå University, Umeå, Sweden; ^4^Umeå School of Sport Sciences, Umeå University, Umeå, Sweden; ^5^School of Sports Sciences, UiT The Arctic University of Norway, Tromsø, Norway; ^6^Department of Community Medicine and Rehabilitation, Geriatric Medicine, Umeå University, Umeå, Sweden; ^7^Department of Community Medicine and Rehabilitation, Section of Sports Medicine, Umeå University, Umeå, Sweden

**Keywords:** home-based exercise, online training, elderly, e-health, ageing, functional strength, sarcopenia, motivation

## Abstract

**Background:**

Physical inactivity and low muscle mass are risk factors for falls, fractures and overall poor health. However, physical activity is reduced with increased age and only a fraction of older adults engages in resistance training (RT). Thus, strategies that facilitate RT among older adults are needed. The aim of the present study was to evaluate the effectiveness and user experience, and explore barriers and motivators toward an online delivered, home-based RT program in older adults with low muscle mass.

**Methods:**

Thirty men and women, 70–71 years of age with low muscle mass were assigned home-based RT with online exercise videos (3 times/week, 45 min/session for 10 weeks) accompanied with an initial supervised try-out session. Quantitative outcome measures included changes in lean body mass and physical function. Semi structured one-to-one interviews with a subset of the participants (*n* = 8) were also conducted to generate a greater understanding of the participants experience of the digitally supported RT. The material was transcribed and analyzed with qualitative content analysis.

**Results:**

Twenty-seven participants (90%) completed the trial. Lean body mass increased by 0.39 kg (95% CI: 0.06–0.72, *p* < 0.05) and chair stand time improved by 1.6 s (95% CI: 0.8–2.3, *p* < 0.001). No significant improvements were seen for balance or gait speed. The theme “*Engaging in Digital Resistance Training with Personal Adaptation Leads to a Sense of Strength and Vitality*” captured the participants experience of the intervention, where a sense of how the body was changing toward a more active lifestyle was described. Instructions, feedback, and intrinsic motivation were identified as key elements for compliance.

**Conclusion:**

The online delivered RT program for older adults with low muscle mass was feasible based on high compliance, user satisfaction, increased lean mass and improved chair-stand time. The participant experiences may explain the high compliance to the intervention and effects on outcomes. Based on these results, online delivered RT could be an accessible exercise routine for older adults with low muscle mass. More research is needed to verify the present findings and assess changes in a long-term perspective.

## Introduction

The world’s population is getting older and physical activity is decreasing with age (Sander et al., 2015). Physical inactivity is now identified as the fourth leading risk factor for global mortality (Dumith et al., 2011). Today, many older adults suffer from sarcopenia, characterized by low muscle mass and low physical function. In fact, the prevalence is as much as 50% among individuals over the age of 80 (Burton and Sumukadas, 2010). Low muscle strength and muscle mass can have a great impact on older adults everyday life as in has been found to associate with physical disability and functional limitation (Hairi et al., 2010), and independently predict falls, fractures, mortality, and overall poor health (Beaudart et al., 2017).

It has previously been shown that brief, whole body resistance training (RT) performed two to three times per week could increase strength and muscle mass and hence decrease the risk of sarcopenia and its comorbidities (Winett et al., 2009; World Health Organization, 2018) and a preservation of a more independent lifestyle (Spirduso and Cronin, 2001). As such, RT at least two times a week is recommended as a part of the physical activity guidelines for older adults (World Health Organization, 2010). However, despite this recommendation, the prevalence of older adults performing RT is low. A national health study from United States showed that only 10–15% of people 55 years and older reported performing any muscle-strengthening activities (Winett et al., 2009).

Supervised RT in “lab-gyms” demonstrate great health effects in older adults (Seguin and Nelson, 2003; Tan et al., 2018). However, when the supervised training program is completed, difficulties in maintaining the training arise (Winett et al., 2009). Barriers regarding RT in older adults include health issues, pain, and lack of time, knowledge, social support and available exercise facilities, whereas common motivators for people to participate in RT include building muscle, falls prevention and feeling more alert (Burton et al., 2017). Collectively, a myriad of motivational factors, both intrinsic and extrinsic, may explain the pursuance of RT or lack thereof. Therefore, strategies that target such barriers and emphasize enablers are likely needed for the promotion of RT and lasting behavioral change. Supervised digitized training could be one solution and previous studies have shown good results in increasing physical outcomes using a tablet-based application (Baez et al., 2017). Less studies have been done on home-based RT with digitized support with the aim to increase muscle mass and function. We have previously examined the effects of a supervised RT program in men and women 70 years of age with pre-sarcopenia (Vikberg et al., 2018). The study showed a significant increase in muscle mass and function, compared with the control group. Such instructor-led training is regarded as golden standard, but it is not cost efficient nor feasible on a community level. As the digital literacy in Sweden is high with 96% of the population being Internet users (The Swedish Internet Foundation, 2020) and older adults in Sweden are among the most frequent Internet users in Europe (Konig et al., 2018), digitized exercise interventions to this population could be an attractive alternative.

The overall objective of this feasibility study was thus to explore the effectiveness and describe user experience including motivational factors of a RT program, delivered as an unsupervised, 10-week long, online training program in men and women, 70–71 years of age with pre-sarcopenia. We hypothesize that the online delivered RT program improves muscle mass and function in older adults with pre-sarcopenia.

## Materials and Methods

### Study Design

This one-armed feasibility trial employed a sequential explanatory mixed-method approach, aiming to evaluate the effects on muscle mass and physical function of a 10-week long, online delivered RT program and to describe participants’ experiences after such training. Assessment was performed at baseline the week before the start of the intervention and then again the week after the intervention was completed. Quantitative outcome variables included body composition, muscle strength, and functional mobility. In order to explore participants experiences of the training, semi-structured, one-to-one interviews were conducted with a subset of the participants after the training program was completed.

### Ethical Criteria

Written informed consent was obtained from all participants prior to the start of the intervention. Participants that participated in interviews were provided with written and verbal information outlining the aims and methods of the interview. The information statement outlined that participation was voluntary and that all contributions would remain anonymous. Consent for the interview was obtained with a signed and dated written form which outlined that participants would be interviewed and audio-recorded. This study was approved by the Regional Research Ethical Review Board, Dnr 2017-132-31M with extension (2018-407-32M).

### Participants

The individuals (*n* = 34) eligible for the present study constituted the control group in a previous randomized controlled trial that examined the effects of a 10-week instructor-led resistance exercise program that aimed to improve physical function and muscle mass in community dwelling men and women aged 70 years (Vikberg et al., 2018).

### Sample Inclusion/Exclusion Criteria

The inclusion criteria to participate in the original study and thus also the present study was based on normative values of muscle mass, in accordance with the pre-sarcopenia definition of the European Working Group on Sarcopenia in Older People (EWGSOP) (Cruz-Jentoft et al., 2010). Specifically, the participants were defined as pre-sarcopenic based on results from a iDXA (Dual-energy X-ray Absorptiometry, GE Healthcare Lunar, Madison, WI, United States) scan when they attended a health examination addressed to all 70-year-old individuals living in Umeå Municipality, northern Sweden (Johansson et al., 2015). The exclusion criterion was not having Internet access and thus not access to the online delivered training.

### Intervention Characterization

All participants (*n* = 30) received an online delivered training program using the same exercises and set-up as a previous successful (supervised) training program for elderly with low muscle mass (Vikberg et al., 2018). The program constituted three training sessions of 45 min a week for 10 weeks. The aim was to offer an identical training program for the home-based training group with digitized support as the intervention group in the previous study had received.

The design of the RT was tailored to increase participants’ muscle mass and physical function. By using the Borg CR-10 scale (Borg et al., 1987; Capodaglio, 2001), moderate to high intensity was instructed to the participants. The training program, consisting of eight sets of whole body exercises including an optional backpack as extra weight and a suspension band for support has been described in more detail previously (Vikberg et al., 2018) and are available online.^1^ For the online delivered training program, one physiotherapist demonstrated the weekly training programs along with a 70-year-old man demonstrating the individual exercises including how to progress and adapt the exercises. The online delivered training included ten different videos, one for each week, with progression including number of sets, encouragement to add weight in backpack and increase the difficulty level of the exercises. The first video included information regarding safety, progression, intensity and structure of the program along with general health benefits of RT in relation to low muscle mass. The following nine videos started with specific new information for the coming week, followed by the exercises. All videos were pre-recorded, uploaded, and accessed online *via* a homepage. During the intervention, support *via* email were available for participants, as members of the research team answered questions continuously. An optional nutritional drink (21–30 g protein, 10–19 g carbohydrates and 1.5 g fat, Gainomax Protein Drink, Norrmejerier, Umeå, Sweden) was also provided to the participants with instructions to drink after each training session to ensure sufficient protein availability to participants for the duration of intervention. The training program was recorded using a Nikon D5600 digital camera and edited using Corel VideoStudioX8 (Corel Corp., Ottawa, ON, Canada) on a Lenovo Ideapad Y700 (Lenovo Group Ltd., China) and uploaded to a website freely available to the participants. For instructional videos of exercises including progression/adaptation and weekly training programs (see text footnote 1).

Before the start of the online delivered program, participants were invited to a one hour long supervised group session with focus on how to perform the exercises, individualize them if needed, and how to use the suspension band and backpack. There was also time for questions regarding the program. During this session, the safety aspect was considered, both technical-wise and how to adapt the training to their own environment, in order to create good conditions for the participants to perform the training. After that session, all participants were demonstrated by AH how to access the online training program and provided with a suspension band and a protocol in where they were instructed to document each training session by checking a box (three preprinted boxes per week, 30 boxes in total).

### Assessments

All assessments were performed at a development and research clinic in the northern part of Sweden, Umeå. Effectiveness of the intervention was assessed on changes in physical function and body composition.

#### Physical Function

To measure physical function, including muscle strength and functional mobility, three different physical performance tests were assessed. The primary outcome was the Short Physical Performance Battery (SPPB), which is a validated and reliable test when examining physical function in lower extremity in older adults (Mijnarends et al., 2013). SPPB includes three tests, balance, gait speed, and chair stands (five-time-sit-to-stand). Based on criteria according to Guralnik et al. (1994) specific cut-points (score 0–4) were applied. A higher total score (maximum 12) indicates a higher degree of functional strength in lower extremity. The second test assessing physical function was the Timed Up and Go (TUG) test, validated and reliable in testing functional mobility in older adults (Podsiadlo and Richardson, 1991; Mijnarends et al., 2013). As a marker on general body strength (Bohannon et al., 2012), isometric muscle strength was tested using a hydraulic hand dynamometer (Jamar; Patterson Medical, Warrenville, IL, United States) (Dodds et al., 2014). The participants were instructed to use their non-dominant hand, keep the arm at a 90° angle and maintain the elbow close to the waist during the test. The maximum value obtained from two consecutive attempts was recorded.

#### Body Composition Including Muscle Mass

Body height (m) was determined bare foot using a stadiometer (Holtain Limited; Crymych, Dyfed, United Kingdom) and body weight (kg) was measured using a clinical scale (HL 120; Avery Berkel, Fairmont, MN, United States). Body composition was assessed using the Lunar iDXA device (Hind et al., 2011). Lean body mass was calculated by excluding fat mass and bone mass from total mass. Appendicular lean mass index (ALMI) was calculated by dividing lean mass from arms and legs from the iDXA scan with height, expressed as kg/m^2^. Total fat mass (FM) was also derived from the iDXA scan (Meredith-Jones et al., 2018).

### Interviews

All participants that completed the training (*n* = 27) were asked if they wanted to partake in individual interviews and 16 participants accepted and were thus eligible for interview. Reasons for non-participation were not explored. The final convenience sample consisted of 8 participants (4 females; 4 males) aged between 70 and 71 years. The interviews took place at the same research and development clinic over a 2-week period in April 2018.

#### Experience of the Training

**FIGURE 1:**
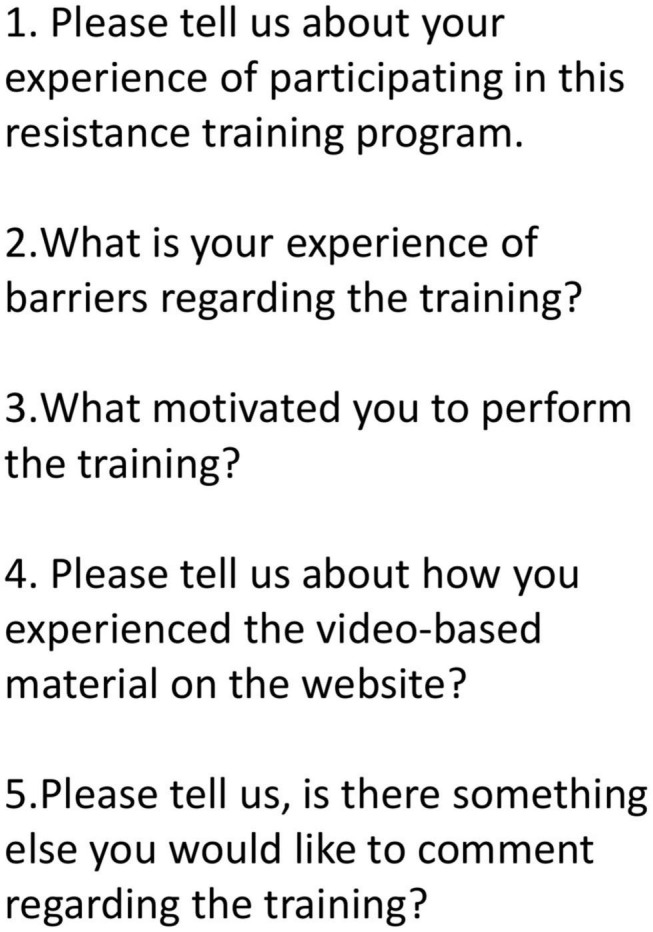
Interview guide illustrating the questions of the interviews.

Interview data were collected through semi-structured, individual face-to-face interviews. The interviews were conducted in a private interview room by AH (Ph.D.) and SB (RN, Ph.D.) of which the later have experience of conducting interviews and qualitative methods. SB had no pre-existing relationships with participants and AH had met with the participants once at baseline when instructing all 30 participants on how to access the online RT videos. The duration of the interviews ranged from 30 to 50 min. An interview guide was used to encourage narration (Sechrist et al., 1987). The interview guide (Figure 1) included questions on participants experience of the training program, motivational factors and on barriers to training. Additional clarifying questions, e.g., “Could you please tell us more about?” and “What did you do then?” were asked in order to get the interviewee to elaborate their response. AH and SB were both present in the room and took turns leading the interview and taking field notes. All interviews were audiotaped. The interviews were transcribed verbatim by SV and the transcript was confirmed against the audio for validation. After eight interviews no new information emerged and we decided that we reached what we considered to be saturation (Saunders et al., 2018).

### Data Analysis

Descriptive statistics were presented as means and standard deviations (*M* ± SD) or median and interquartile range (IQR). Shapiro–Wilk test for normality were used to determine if the data was normally distributed along with a visual inspection using histogram. Paired samples *t*-test of pre- and post-intervention values were used for physical function and body composition to assess potential changes over time. Potential differences in baseline and outcome characteristics between the group that was interviewed and the group that was not interviewed were assessed with independent samples *t*-test. All statistical tests were performed using SPSS for Macintosh Version 25 (Armonk, NY, United States: IBM Corp.). Alfa level was set to 0.05 for statistical significance. As the participants of the present trial constituted the control group of a previous trial where supervised resistance training was evaluated (Vikberg et al., 2018), no additional power calculation was made.

Interview-data were analyzed using qualitative content analysis focusing on both manifest and latent content (Graneheim and Lundman, 2004; Graneheim et al., 2017). The transcript was read through several times to obtain a sense of the text. Following that, three authors (AH, SB, and SV) divided the text in to meaning units independently. AH, SB, and SV discussed the meaning units until consensus was reached. Thereafter, the first author (SV) condensed the meaning units and labeled them with codes describing the core content close to the text. Codes were sorted based on similar content into sub-categories. Sub-categories with similar content were sorted into categories AH, SB, and SV discussed the codes, subcategories and categories until consensus was reached (Graneheim et al., 2017). Finally, categories were interpreted and abstracted in to a theme explaining the underlying meaning between the categories (Erlingsson and Brysiewicz, 2017). During this process SV, SB, and AH made comparisons with original text and discussed the results. Microsoft Word was used for transcript, meaning units, condensing meaning units, and coding. The rest of the analysis process was carried out by hand.

## Results

**FIGURE 2:**
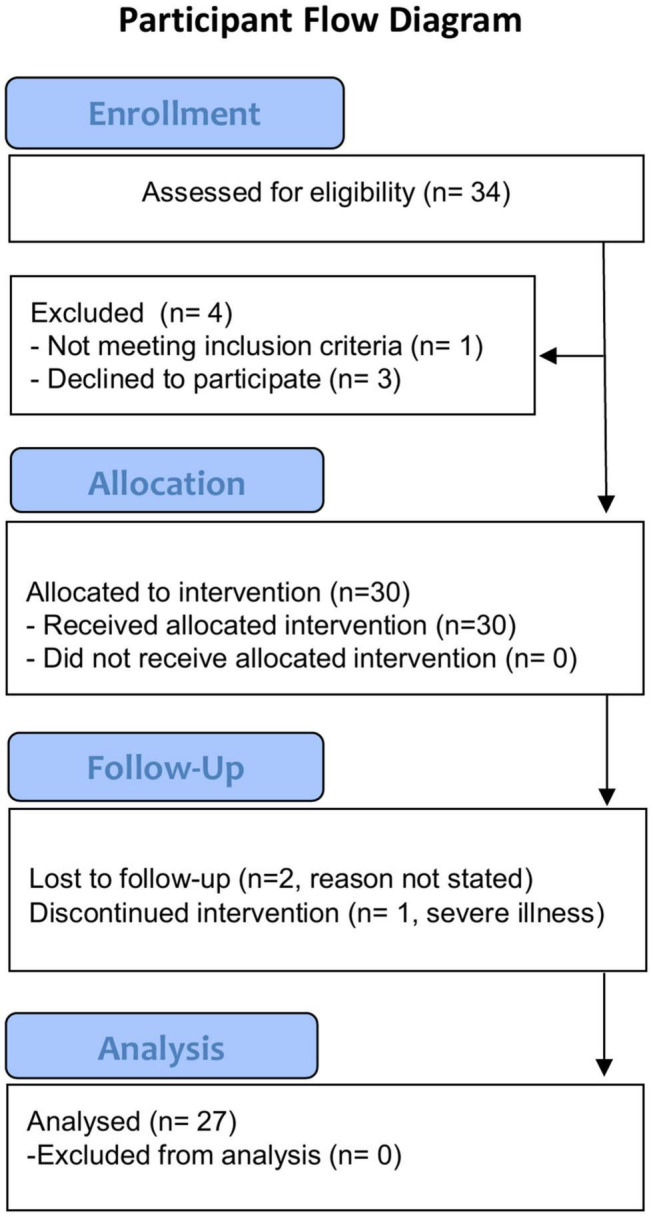
Participant flow overview.

Thirty-four individuals were eligible for inclusion in the present trial [see Figure 2 for CONSORT (Schulz et al., 2010) flow diagram]. Three subjects declined participation before the start of the intervention. The reasons given were acute lumbago (*n* = 2) and knee arthroplasty (*n* = 1). One participant was excluded for not having Internet access, leading to a final sample of 30 participants that were enrolled in the trial (see Table 1 for baseline characteristics). Three people did not complete the study, one due to severe illness (*n* = 1) not related to the intervention and the other two did not state a reason for dropping out. The remaining 27 individuals (90%) all completed the study and eight participants, four men and four women, were subsequently interviewed. The mean self-reported completion rate of the training sessions was 80% (median 29 sessions, IQR 25–30). Baseline characteristics were not significantly different between the group that were interviewed and those who did not partake in interviews, *p* > 0.05 for all.

**TABLE 1:** Participant characteristics at baseline.

Variables	Total *n* = 30
Age (years)	71.1 ± 0.26
Female, *n* (%)	15 (50)
Height (m)	1.71 ± 0.10
Weight (kg)	68.9 ± 13.4
BMI	23.5 ± 3.13

*Values are presented as means ± standard deviations, except where otherwise indicated. BMI, body mass index.*

### Effects on Muscle Mass and Physical Function

When observing changes on physical function, no significant difference was seen for the total SPPB score, although the chair stand time improved by 1.6 s (95% CI: 0.8–2.3, *p* < 0.001; Table 2). No significant changes were observed in the other measures on physical function (balance, gait speed, TUG, and grip strength). For the body composition measurements, ALMI improved by 0.1 kg/m^2^ (95% Cl: 0.02–0.18, *p* = 0.018), total lean mass improved by 0.39 kg (95% CI: 0.06–0.72, *p* = 0.024), leg lean mass by 0.24 kg (95% CI: 0.04–0.43, *p* = 0.018) and total fat mass decreased by 0.32 kg (95% CI: 0.01–0.64, *p* = 0.045; Table 2). No significant differences in any of the outcome measures were observed between the participants that were interviewed and the other participants (*p* > 0.05 for all).

**TABLE 2:** Changes in outcomes on lean body mass and physical function following 10 weeks of digital resistance training.

Training group *n* = 27	Pre-training	Post-training	*p*
**SPPB**			
Gait speed (s)	3.55 ± 1.52	3.57 ± 1.69	0.862
Chair stands (s)	9.46 ± 2.37	7.91 ± 1.98	**< 0.001**
Balance (0–4)	3.81 ± 0.62	3.85 ± 0.53	0.574
Total score	11.44 ± 1.34	11.59 ± 1.42	0.256
TUG (s)	9.14 ± 3.88	9.71 ± 4.84	0.059
Handgrip (kg)	32.90 ± 10.16	33.50 ± 10.39	0.131
**DXA measurements**			
ALMI (kg/m^2^)	6.34 ± 0.88	6.44 ± 0.83	**0.018**
Total lean mass (kg)	43.42 ± 8.20	43.81 ± 8.32	**0.024**
Arm lean mass (kg)	4.82 ± 1.41	4.86 ± 1.38	0.311
Leg lean mass (kg)	14.15 ± 2.98	14.39 ± 2.88	**0.018**
Total fat mass (kg)	22.93 ± 7.83	22.61 ± 7.53	**0.045**

*Values are presented as means ± standard deviation. SPPB, Short Physical Performance Battery; TUG, Timed Up and Go; DXA, Dual-energy X-ray absorptiometry; ALMI, appendicular lean mass index. The bold values indicate a significant change (p < 0.05) between pre-, and post training.*

### Adverse Events and Side Effects During the Intervention

One participant reported difficulties in performing the training program due to a musculoskeletal problem. Another participant reported an injury not related to the intervention; however, it affected the training, and the person could not perform all exercises. One person had difficulties in performing hand grip strength assessment due to arthritis. Pain relief from already existing pain during the intervention, e.g., relieved back pain and shoulder pain were also reported. Delayed-onset muscle soreness was also reported by most of the participants.

### Experience of a Home-Based Resistance Training Program

The qualitative content analysis of the interviews resulted in an overall theme, “*Engaging in Digital Resistance Training with Personal Adaptation Leads to a Sense of Strength and Vitality.”* The theme was based on four categories and fourteen subcategories (Table 3). Representative quotes of each subcategory can be found in Table 4 [quote (Q)1–Q47] and are summarized by each category below.

**TABLE 3:** Categories and sub-categories building the overall theme: “*Engaging in Digital Resistance Training by Personal Adaptation Leads to a Sense of Strength and Vitality*.”

Comprehending the training program	Being committed and making adaptations	Experiencing bodily sensations	Experiencing vitality and wellbeing
Structure of the training program	Flexibility	A stronger body	A desire to be more physically active
Instructions	Routines	Pain/discomfort	Enjoying exercise
Progression	Motivation	Discrepancy between measured- and experienced results	Healthy choices
Utilization of the digital support			Satisfaction

*The overall theme “Engaging in Digital Resistance Training by Personal Adaptation Leads to a Sense of Strength and Vitality” reflects how participants experienced the implementation of the training program and their experiences of their body and functioning.*

**TABLE 4:** Quotations from the interviewed participants categorized according to subcategories. Q = quote.

Structure of the training program	Q1 *“And so, when one were lying on the floor, there were two exercises after each other. So, it wasn’t first up and then down again, that takes time, but it followed the exercises well.”* Q2 *“It (the training) was for the whole body, because, well when you’ve done one thing, it was well thought out, it was a good fit.”* Q3 *“In the beginning it was good to take a break after each exercise. But then [between the exercises] when you had to change equipment, you never had time to fix it. Then it was already up and running. I had not rolled out the mat, for example. There was a need for a break to get things in order.”*
Instructions	Q4 “*they [The instructors] showed how to use the equipment and how to do the exercises. I felt that it was needed.”* Q5 *“She showed the exercises so well and was so good in the first videos. The first few times she gave a lot of information, but then I thought, if she’ll keep on like this, it becomes a bit boring [*…*] but the information in the beginning was needed.”* Q6) “The *introduction was great, it was the start of the whole training, I liked that. It was like getting started. If you just get a paper saying do this and that, it would never work. Thanks to the introduction, where we had the chance to try and see how to perform the exercises, it made it all easier during the time training on your own.”* Q7 *“Instructions were good but getting feedback halfway would have made it even better, to know if I did [the exercises] right for example, and also how I could progress if I needed and adapt [the exercises] to my weaknesses.”* Q8 *“Sometimes I didn’t see everything, for example, if I was lying on the floor. And I thought, the music, it could be clearer when it’s over [the exercise]/*…*/there could have been some signal that now it is over, done.”*
Progression	Q9 *“I borrowed a backpack and filled it with books which I used the last 5 weeks.”* Q10 “*I didn’t think it was that difficult and I thought I couldn’t have more (weight) in my backpack, but when she [the instructor] said I should have more [weight] in my backpack it went very well, it wasn’t that difficult.”* Q11 *“I slightly modified [the training program] toward the end/./when lying down and performing hip thrusters, then I did it on one leg 10 times and on the other 10 times instead of doing with both legs because it gave no resistance. Then I sidestepped the program and did 10 with one leg and went straight to the other leg because then one leg had rested, I thought.”* Q12 *[progression] no I did not, I did not think of it, no I actually did not.*
Utilization of digital support	Q13 *I had my iPad but it did not always work that well. Sometimes I used my telephone instead, to be able to see the time but then I realized it worked better with my computer. When I did 10 repetitions and then rest 60 seconds, sometimes it did not start after the resting period, the Internet connection was not always great. Sometimes I had to start all over again, but it was easier with the computer. I stopped using my iPad because it stopped after three exercises, I was irritated because I wanted to do it all”.* Q14 *“I had a system in the living room that I used with a computer. But that computer was pretty bad and slow, it took time for the video to get started, so I took my laptop instead and it instantly worked well. I set it up and at the end I didn’t need to see anything, I just had it as a timekeeper.”* Q15 *“At the start I used my computer [to watch the videos] and then I used my phone/./additionally, I also wrote down the exercises in order to remember [which exercises] to do.”*
Flexibility	Q16 *“When I went to Stockholm, I could still do my work out. I was visiting my grandchild, I put my training clothes on, and she asked why, I said now I shall do my training exercises, you may do it with me”.* Q17 *“I like this [homebased training] to handle it yourself, to be free, instead of having to go somewhere three times a week.”* Q18 *“I have many friends who go to different places [fitness centers] but this [training program] is also a workout in the same way really, I do not have to go to those places and pay an expensive sum.”* Q19 *“It was one exercise that was scary, with those bands, I was always so scared they [the suspension bands] would let go, so I had an air mattress underneath.”*
Routines	Q20 *“I had it all prepared. I woke up, hade some breakfast, sat in the sofa, then I started the training at 9 am and it was all set. I had my iPad and did everything following the instructions.”* Q21 “*I do not think that [the training program] have been hard to do/./it becomes a routine, that’s what is important.”* Q22 *“I am satisfied with the training, did not think it took up a lot of time.”*
Motivation	Q23 *“I told my sister I was participating in this program and she asked me if I really did the training three times a week and I said of course, I have signed up for this and I want to do it. I told her that I am busy for an hour and I don’t want to be disturbed.”* Q24 *“If I have decided on something, I will do it.”* Q25 *“I Wanted to see the results of the training program and to measure changes.”* Q26 *“I really wanted to change myself.”* Q27 *“I would like to feel that my body is younger [than my actual age].”* Q28 *“It was like having a training friend when watching the videos, because it was the same girl showing the introduction training, it felt like we met again.”* Q29 *“due to the fact that I was able to see [the video] how to do it [the exercise] then you also had like a friend with you which you met again [each training session].”* Q30 *“Me and my husband have been training together in a room at home.”*
A stronger body	Q31 “I have always liked to be active, but now I wanted to get more upper body muscles * laugh * again, I look so skinny. I have gained a little more muscle, I have, but not so much that I would like, not because I want to become a body builder, but still.” Q32 *“I have gained a firmer body and, above all, become stronger in my legs.”* Q33 *“and then I have become a little stronger//.//in the legs if nothing else”* Q34 *“I seem to have gotten a little stronger/./I feel lighter.”* Q35 *“The hardest exercise was push-ups. I started off on my knees but in the end I did push-ups on my toes.”*
Pain/discomfort	Q36 *“It [push-ups] Induced pain in the neck area and created terrible tension in the entire body.”* Q37 *“I could not do push-up on the floor, It caused me pain so I did it against the wall instead.”* Q38 *“I haven’t felt pain at all even though I’ve suffered from back pain during my whole life.”*
Discrepancy between measured and experienced results	Q39 *“I feel good, I did this for my own good and I have been wanting to start working out for a long time. However, my result from the body composition scan was bad, it doesn’t really matter, but I thought it was strange, it should have been better, I was a bit disappointed.”* Q40 *“for me it was strange, I had almost not gained any muscle mass but my fat mass had decreased, a few kilos but*…”
A desire to be more physically active	Q41 *“I now feel that I want to move my body. I want to go out and walk and I am longing to do the exercises because of the reward I get afterwards.”* Q42 *“I have even thought oh, what if I could start jogging, at least a little bit, and I think that maybe I can, I have gone out and bought new shoes.”*
Enjoying exercise	Q43 *“I feel happiness in my body when I use it [my body], I have never felt so good. The more active I am, the better health I achieve. I have diabetes, all tests improve with training. I need to watch out for the down days, the best is to stay active and a positive thing is that I now like it, I feel good about it.”*
Healthy choices	Q44 *I somehow think I feel more alert. When I go to my friend, I always take the stairs and before I always took the elevator. I like it a lot, it is like I have become more alert.”* Q45 *“Yes, knowing that you have been active and partly that you are not just sitting in front of the TV or doing something else. It feels good afterwards, that you did it, anyway, and activate yourself a bit.”*
Satisfaction	Q46 *“I was a lazy person before. I am very satisfied that I’ve manage this training.”* Q47 *“If I had anything else planned, do I have time to [work out]? When I was stressed out. but no, then after I completed it [the training], it felt so good.”*

#### Comprehending the Training Program

This category was formed from four sub-categories (*Structure of the training program, Instructions, Progression*, and *Utilization of the digital support*; Table 3).

Participants expressed the importance of the structure of the program being easy to follow (Q1 and Q2). Having time between the different exercises to be able to prepare for the next exercise, similar exercises following each other (Q3) and acoustic indication to clarify the breaks was described as facilitating the training.

Clear instructions were important; most of the participants were satisfied with the instructions and some wanted more. Also, being instructed how to use the equipment along with watching how to perform the exercises was of high value (Q4 and Q5). The in-person introduction was highly appreciated and the opportunity to learn the exercises and ask questions were emphasized (Q6). Some participants expressed that feedback during the intervention would have been appreciated (Q7). Not having enough competence in how to perform the exercises and being unsure if the execution of the exercises were correct created insecurity (Q8).

Participants described that the progression in the training program was good. However, the way participants progressed in the program varied. Some of them did the most difficult exercise from the start and added weight during each week, whereas others did the easiest level of difficulty and added weight in the backpack as progression (Q9 and Q10). Others found it too hard to add weight and instead increased the number of repetitions (Q11). Other participants did not increase the difficulty level (Q12).

For the utilization of digital equipment, most participants described it as easy to use while a few expressed some difficulties. Sometimes the video did not work, not knowing if it had to do with the Internet connection or the hardware, but for most participants it worked well (Q13 and Q14). Some participants described how they first watched the videos, then wrote down the exercises, to be prepared for the next exercise to come (Q15).

#### Being Committed and Making Adaptations

This category was formed from three sub-categories (*Flexibility, Routines*, and *Motivation*; Table 3).

The flexibility of performing the training program whenever and wherever was appreciated by participants (Q16 and Q17). Participants also narrated on making a personal adaptation of the training and preferred training at home for economic reasons (Q18 and Q19).

Assimilating the training program into their daily routines were expressed as a good strategy in order to get the training done (Q20–21). Participants did not find the training time consuming (Q21 and Q22).

Participants narrated on the importance of taking responsibility for the training (Q23) and they also underlined that older people keep their promise. Participants also emphasized the importance of setting their own goals to keep the motivation high and perform the training properly (Q24). Common goals were building muscles and wanting to see a difference in the results of the body scan in the end of the training program (Q25 and Q26). Some participants also expressed that they wanted to look younger, healthier and become more fit in general (Q27). Participants described feelings of affinity seeing the same instructor in the video each time and that the instructor became a training buddy (Q28 and Q29). Some participants also involved their spouse or friends in the training (Q30).

#### Experiencing Bodily Sensations

The category was formed from three sub-categories (*A stronger body, Pain/discomfort*, and *Discrepancy between measured- and experienced results*; Table 3).

Participants experienced a range of physical improvements from taking part in the RT program. These included feeling stronger, lighter, and in less pain. They mentioned discovering a bodily change and heightened awareness of their bodily sensations (Q31). Participants experienced improved body strength (Q32–35) and narrated on specific moments in daily life when they had experienced their new strength such as that biking up the hill was easier after the period of RT than prior to the intervention.

Participants experienced sore muscles predominantly during the first weeks of the training program. Some exercises were more prone to cause pain and discomfort, especially push-ups which were perceived as hard to perform, partly due to shoulder pain (Q36–37). On the other hand, relief of chronic pain was also perceived as a consequence of the intervention (Q38).

Some participants were disappointed with the measured outcome results and were uncertain what the result meant when comparing the objective measures with their perceived bodily changes (Q39 and Q40).

#### Experiencing Vitality and Wellbeing

This category was built from four sub-categories (*A desire to be more physically active, Enjoying exercise, Healthy choices*, and *Satisfaction*; Table 3).

A new desire to be more physically active were frequently mentioned by interviewees. Participants mentioned that the training helped to generate new energy and a willingness to partake in the training. Participants expressed a sense of joy toward exercising, for instance now being more prone to taking the stairs while always choosing the elevator before the intervention. They described how they now were longing to live a more physically active life and how good it felt in their body after exercising, a feeling they have not felt before (Q41 and Q42).

Participants narrated on how they had started to pay more attention to themselves and that exercising had led to a healthier life, they expressed a sense of happiness toward training (Q43). Healthier choices and lifestyle changes were perceived as a positive outcome of the training. They had improved both their physical and mental health and narrated on how much better it felt performing a training program compared with watching TV (Q44 and Q45). Some spoke of themselves as previously being lazy and expressed happiness to be able to maintain the training program and a willingness to go on with training (Q46 and Q47). Performing the training was also seen as a challenge and participants described a feeling of being satisfied with-, and proud of themselves for completing the training.

## Discussion

In this feasibility study we examined the effectiveness and the user experience of an online delivered RT program for older adults with pre-sarcopenia. The main results were that the easy-to-use, digitized RT program increased muscle mass and chair-stand time in this population with low muscle mass while, at the same time, being feasible based on high compliance and participant satisfaction. Furthermore, the information from the interview data strengthens the objective outcomes as the participants experienced both a physical and mental change where they felt more alert, lighter and expressed a desire to live a more active life.

According to previous studies, the compliance in home-based training is usually low (Argent et al., 2018). Therefore, in the present study, the completion rate of 90% as well as the high adherence rate to the training sessions (80%) was very appealing. Only minor adverse events were reported, and participants even reported relief of chronic pain as a consequence of the intervention. The use of the digital platform worked well for most of the participants, possibly partly related to a high digital literacy and a widespread information and communication technology infrastructure in Sweden (Konig et al., 2018; The Swedish Internet Foundation, 2020). However, some mentioned that they also wanted to write down the exercises, mainly, according to the participants, to be prepared for the next exercise. Participants were pleased with the instructions given but wanted more feedback during the program. This indicates that closer follow-ups could improve the compliance of the training even further as the requested feedback can be related to extrinsic motivation, an important motivational factor in early stages of behavioral change (Thogersen-Ntoumani and Ntoumanis, 2006). The instructions given in the training videos together with the try-out session at the start of the intervention was highly appreciated and some stated that it gave them confidence in knowing that they performed the exercises in a correct manner. Furthermore, as the try-out session was designed as a group activity, it gave the participants a chance to meet each other as a group and share experiences and expectations. Other aspects of relatedness described by the participants was perceiving the training instructor in the videos as a training friend and including a spouse or a grandchild in the training sessions. Moreover, the training program was designed in a flexible way so that it could be performed anywhere, anytime as long as there was an Internet connection and a place to attach the suspension bands. Although we did not instruct the participants to train in a certain time or day it was clear that incorporating the training into their daily routine was an important factor in order to get the training done. Together, the above-mentioned examples could be seen as aspects of competence, relatedness and autonomy, all key components in creating intrinsic motivation according to the self-determination theory (Teixeira et al., 2012). In light of the self-determination model and previous studies (Thogersen-Ntoumani and Ntoumanis, 2006; Mehra et al., 2016), these aspects could in turn be interpreted as facilitators for exercise. These are important findings that, if targeted, could aid in overcoming some of the previously described barriers to RT in older adults (Burton et al., 2017). In the present study, a feeling of overcoming some of these barriers were expressed. People reached a level of autonomy in terms of finding joy toward exercising and a willingness to go on with the training. The examples of making healthy choices and possibly even long-term lifestyle changes as a positive side effect of the training program pointing in a direction of overcoming barriers toward living a more active life.

According to previous studies, RT is known to increase muscle mass and function in older adults (Marcos-Pardo et al., 2018), especially in “lab-gyms” (Seguin and Nelson, 2003; Tan et al., 2018). In the present study, the effectiveness of the RT was assessed from body composition and physical function measures. Total lean body mass, leg lean body mass and chair-stand test did all significantly improve after 10 weeks of home-based training, albeit not as much as was seen in the instructor-led training intervention conducted earlier (Vikberg et al., 2018). These results are in accordance with a study comparing group-based and home-based exercise for older adults with sarcopenia who found that group-based training resulted in larger health improvements than home-based training (Tsekoura et al., 2018). One possible explanation to the observed difference in results between supervised and unsupervised training could be that not all participants progressed the difficulty level and resistance load for the exercises according to instructions, which is a common draw-back of unsupervised exercise programs for older adults (Lacroix et al., 2017). Regarding the measures of physical function, relatively small effects on SPPB were observed, which can be explained by a ceiling effect (mean baseline score 11.7 of a maximum of 12). Such ceiling effect could have been avoided by using tests such as 30 s chair stand test (Jones et al., 1999) or the continuous-scale physical functional performance test (Cress et al., 2005), instead of SPPB. Also, as both the gait test and TUG test instructs the participants to adhere to their normal walking pace, they may not necessarily reflect the participants maximal capacity or change thereof. Still, we found a significant improvement in the five-time sit-to-stand test that is part of the SPPB which corresponds to the minimum detectable change of 17% according to a previous study examining chair stands in older women (Goldberg et al., 2012). Some of the interviewed participants that had not improved their physical function and muscle mass during the outcome assessments felt a dissonance as they still felt stronger than before the intervention. Relying on feedback can be related to extrinsic motivation and participants also described a curiosity of the results and narrated on being disappointed when results were not in line with their experience of the training. However, it was noticeable that participants also expressed a desire to exercise, making healthier choices in their everyday life and the importance of having routines for training. This could be interpreted as having intrinsic motivation and that the participants had reached some degree of autonomy according to the self-determination model (Thogersen-Ntoumani and Ntoumanis, 2006; Teixeira et al., 2012).

As the world’s population have a steadily increasing life expectancy and 50% of persons over the age of 80 have been shown to suffer from sarcopenia (Burton and Sumukadas, 2010; Heath et al., 2012), early prevention of sarcopenia should be a priority. Our recently conducted study showed that supervised training increased muscle mass and improved chair-stand time in men and women with low muscle mass (Vikberg et al., 2018), properties that are associated with a reduced risk of falls, fractures, and death (Denison et al., 2015). However, instructor-led training is not cost-efficient nor feasible on a community level. Therefore, there is a need to evaluate other, more available measures of prevention for older adults at risk of sarcopenia, with the aim of reducing healthcare costs and improving quality of life for older people (Malafarina et al., 2012). As such, the results from this study could increase the knowledge and aid in promoting guidelines on how to design training programs and how to maintain compliance in RT for older adults, without the need of gym equipment or exercise facilities. However, one important consideration when implementing digitized interventions is the digital literacy of the study population and the information and communication technology infrastructure of the region of interest.

The strength of this study was the use of a mixed method approach, where the qualitative results strengthen the quantitative data and together generates a more complete picture of the feasibility of the study. Another strength of the intervention was the easy-to-use training program which did not require access to gym-equipment yet still allowing individual adjustment both regarding progression and intensity while at the same time focusing on reducing risks and adverse events. Limitations of the study includes the subjectivity of the intensity level, which is difficult to control even though the participants had an introduction session learning how to apply the intensity to their training. If more instructions and feedback during the training would have been given, maybe the results would have improved even further. Further, we did not document which of the participants that utilized the optional nutritional drink that was offered to all participants, so we cannot evaluate its potential effect on lean mass gain. Another potential limitation is that the participants that did not participated in the interviews had different experiences of the training program compared with those who accepted to be interviewed. The sample size is another potential limitation regarding the quantitative results of the present study, as is the absence of a control group. A larger sample would have increased the statistical power of the analysis and potentially influenced the results. Finally, a follow-up assessment of the participants would have been valuable to evaluate long-term effects of the intervention.

## Conclusion

An online delivered easy-to-use home-based RT program led to increased muscle mass and improved chair-stand time in older adults with low muscle mass. The user experience of the digital training program included personal adaptation and a sense of competence, health, and well-being. Furthermore, were identified instructions, feedback, and intrinsic motivation as key elements for compliance. Together, these findings suggest that it is feasible to conduct this type of online delivered RT program in community-dwelling older adults with low muscle mass. However, more research is needed to verify the results and assess long-term changes.

## Data Availability Statement

The raw data supporting the conclusions of this article will be made available by the authors, without undue reservation.

## Ethics Statement

The studies involving human participants were reviewed and approved by the Regional Research Ethical Review Board, Dnr 2017-132-31M with extension (2018-407-32M). The patients/participants provided their written informed consent to participate in this study.

## Author Contributions

SV, SB, AN, PN, and AH: study concept and design, and analysis and interpretation of data. SV, SB, and AH: acquisition of data and drafting of the manuscript. All authors read and approved the final manuscript.

## Conflict of Interest

The authors declare that the research was conducted in the absence of any commercial or financial relationships that could be construed as a potential conflict of interest.

## Publisher’s Note

All claims expressed in this article are solely those of the authors and do not necessarily represent those of their affiliated organizations, or those of the publisher, the editors and the reviewers. Any product that may be evaluated in this article, or claim that may be made by its manufacturer, is not guaranteed or endorsed by the publisher.

## Footnotes

1 https://www.youtube.com/playlist?list=PLrpt_1yETsoKzEBh6oAFMRUWfwbVJ5Reo. Legends are in Swedish, *Övning 1* = exercise 1 and *Styrketräning Vecka 1* = resistance training week 1.

## Abbreviations

ALMI: appendicular lean mass index
BMI: body mass index
DXA: Dual-energy X-ray Absorptiometry
RT: resistance training
SPPB: Short Physical Performance Battery
TUG: Timed Up and Go.
